# Phase Contrast Imaging Reveals Low Lung Volumes and Surface Areas in the Developing Marsupial

**DOI:** 10.1371/journal.pone.0053805

**Published:** 2013-01-18

**Authors:** Shannon J. Simpson, Karen K. W. Siu, Naoto Yagi, Jane C. Whitley, Robert A. Lewis, Peter B. Frappell

**Affiliations:** 1 University of Tasmania, Hobart, Australia; 2 Centre for Synchrotron Science, Monash University, Victoria, Australia; 3 SPring-8/JASRI, Sayo, Hyogo, Japan; 4 Department of Primary Industries, Attwood, Victoria, Australia; Vanderbilt University Medical Center, United States of America

## Abstract

Marsupials are born with immature lungs when compared to eutherian mammals and rely, to various extents, on cutaneous gas exchange in order to meet metabolic requirements. Indeed, the fat-tailed dunnart is born with lungs in the canalicular stage of development and relies almost entirely on the skin for gas exchange at birth; consequently undergoing the majority of lung development in air. Plane radiographs and computed tomography data sets were acquired using phase contrast imaging with a synchrotron radiation source for two marsupial species, the fat-tailed dunnart and the larger tammar wallaby, during the first weeks of postnatal life. Phase contrast imaging revealed that only two lung sacs contain air after the first hour of life in the fat-tailed dunnart. While the lung of the tammar wallaby was comparatively more developed, both species demonstrated massive increases in air sac number and architectural complexity during the postnatal period. In addition, both the tammar wallaby and fat-tailed dunnart had lower lung volumes and parenchymal surface areas than were expected from morphometrically determined allometric equations relating these variables to body mass during the neonatal period. However, lung volume is predicted to scale with mass as expected after the neonatal marsupial reaches a body mass of ∼1 g and no longer relies on the skin for gas exchange. Decreased lung volume in the marsupial neonate further supports the maxim that cutaneous gas exchange occurs in the marsupial neonate because the respiratory apparatus is not yet capable of meeting the gas exchange requirements of the newborn.

## Introduction

Marsupials are born with structurally immature lungs by eutherian standards. The fat-tailed dunnart *(Sminthopsis crassicaudata*) [1] and quokka wallaby (*Setonix brachyurus)* [2] are born with lungs at the canalicular stage of development, though more commonly marsupials, including the tammar wallaby (*Macropus eugenii*), are born with lungs at the saccular stage [3]. Consequently, the majority of lung development in marsupials occurs in air in the pouch environment.

The lung of the newborn tammar wallaby contains abundant surfactant [4], [5] and is largely functional as a gas exchanger, contributing 60–70% of the gas exchange required to meet the total metabolic demands of the newborn [6]. The remaining 30–40% of gas exchange is cutaneous, with the skin continuing to play a role in meeting metabolic demands until four days postpartum (P4) in the tammar wallaby [6]. Surfactant is also present in the smaller newborn dunnart species [1], yet in these species reliance on the skin for gas exchange approaches 100% at birth [7], [8], [9]. In the fat-tailed dunnart the skin contributes to gaseous exchange until P40 [1], enabled in part by the large surface area to volume ratio associated with a very small body size, low metabolic rate, possible neural and/or mechanical constraints to pulmonary ventilation and the presence of cardiac shunts in these immature newborns [7].

Traditional morphometric techniques have established that throughout development lung volume scales isometrically, and identically, in marsupials and eutherians [10], [11], [12]. However, on a mass-specific basis the quokka appears to have a relatively small lung volume at birth while the tammar wallaby and the North American Opossum (*Didelphis virginiana*) have larger than predicted mass-specific lung volumes [7].

Phase contrast imaging enhances edges of material boundaries, where differences in refractive index occur, for example the boundaries between different tissue types [13]. A marked phase contrast exists between the air in the lungs and the surrounding tissues, making this method particularly good for high definition imaging of the airways. As a result, phase contrast imaging with a synchrotron radiation source has been used to elucidate the biomechanical mechanisms of tracheal compression, and the role of convective respiratory mechanisms in a range of insects [14], [15], [16], [17]. Phase contrast imaging has also been used to provide further insight into the alveolarisation of mouse lungs [18], [19] and the clearance of fetal lung liquid during the first breaths in rabbit pups [20], [21]. Until recently, synchrotron imaging studies of the lung often focused on methodology [22], [23], [24], [25], [26], [27], and have always been undertaken in mammals which have comparatively more mature lungs than the newborn marsupial.

This study aimed to explore lung development in the tammar wallaby and fat-tailed dunnart during the first weeks of life using phase contrast imaging with a synchrotron radiation source. In addition, we aimed to calculate functional lung volumes and surface areas during development using three-dimensional (3-D) reconstructions of computed tomography (CT) data.

## Materials and Methods

### Animal collection

Tammar wallabies and fat-tailed dunnarts from a laboratory colony were continuously monitored for signs of giving birth, and/or underwent regular pouch checks when approaching full-term (26–28 days and 13–14 days [28] respectively). The time of birth was noted to within an hour for each pouch young utilised in a time point less than 3 days, while the day of birth was noted for those utilised at time points greater than 3 days. Young ranging from birth to 20 postnatal days in the case of the fat-tailed dunnart and 30 postnatal days in the case of the tammar wallaby were collected, weighed and euthanized by cold narcotisation and anaesthetic overdose with halothane under animal ethics permit (LTU AEC 04/37(L)), approved by La Trobe University Animal Ethics Committee.

### Sample preparation

Once killed (generally less than one minute), animals had their mouth and nares blocked with a quick setting polyether compound (Impregum F, ESPE) to aid in prevention of air-loss and were immediately frozen on dry ice prior to being stored at −20°C in an evacuated zip lock bag with moist gauze to prevent desiccation. Specimens were maintained at −20°C until they were put on dry ice for air-transport to the Biomedical Imaging Centre, SPring-8 synchrotron, Hyogo, Japan. For imaging, pouch young were placed into a snug fitting plastic tube and held in place with a plastic pin that prevented movement during imaging. A cryostat was placed in the experimental hutch within the centre and a cold stream of air was directed over the specimen to ensure the sample was kept just above the freezing point, ensuring that ice crystals did not interfere with imaging.

### Phase contrast imaging

Phase contrast enhanced single images and computed tomography data sets were acquired. To provide maximum edge enhancement to elucidate the lung structure, single images were acquired on the high coherence 20XU beamline, using a propagation distance of approximately 3.7 m and an X-ray energy of 17.7 keV. Images were recorded using a Hamamatsu phosphor charged-coupled device with pixel size of approximately 3×3 µm^2^. Since the beam size is limited (approximately 3 mm vertically and 6 mm horizontally) on the 20XU beamline, several images were taken in a raster scan and then tiled together to create the final image in animals that were larger than these dimensions. Images were conventionally corrected for dark current offset and full field non-uniformities before tiling (if required) [29]. A larger beam size is available on Beamline 20B2 (approximately 25 mm vertically and >300 mm horizontally) and this beamline was used to acquire computed tomography data at 20 or 25 keV. A shorter propagation distance of 15–60 cm and higher X-ray energy was used to avoid tomography reconstruction artefacts. Between 1296 and 3772 projections were taken over 180° around the axis of rotation, depending on the step size. The exposure time was 1000 or 1500 ms for each projection. Since the synchrotron beam is essentially parallel, computed tomography reconstructions were carried out using a first generation CT geometry on a slice by slice basis, leading to over 1000 transverse slices being reconstructed.

### Lung surface area and volume calculations

Lung surface areas and volumes were calculated using ImageJ (National Institutes of Health). Firstly, each set of reconstructed images was thresholded (from grey scale) to create a binary image that could be easily segmented to highlight lung regions only. The images were then inverted so that the lungs appear white, and non-air filled structures are black, which is necessary for the morphological filters to run. These filters reduce any noise in the images. Volumes and surface areas were then calculated for each CT slice by using the “analyse particle” function which finds regions in the binary images between certain sizes (>30 and <3500 pixels) and counts their number, area and perimeter. Surface areas were calculated by summing the perimeter for each slice and the volume determined by summing the area and knowing the thickness of each slice was 1 pixel.

### Allometry

Where appropriate, least-squares regression was fitted to log-log transformed data for allometric analysis. The equality of the slope of the regression lines for marsupials collected in this study was assessed using a parallel line analysis. All data analysis was performed in SigmaPlot 12 (Systat software, USA). Significance was considered at p<0.05.

## Results

**Figure 1:**
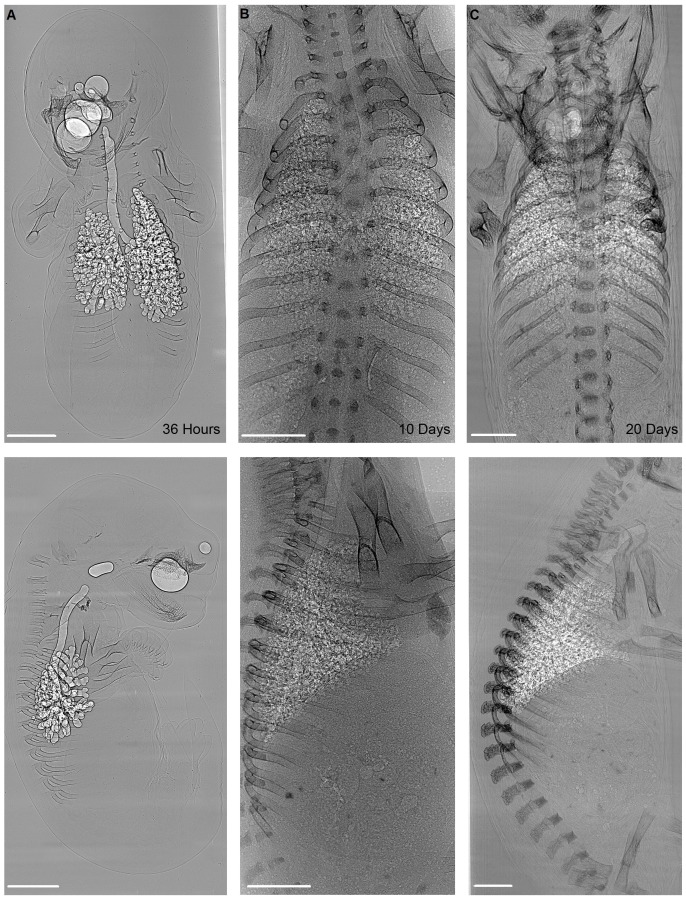
Phase contrast X-ray imaging of the developing tammar wallaby using a synchrotron radiation source. Anterior-posterior (top) and lateral (bottom) views of tammar wallaby pouch young aged 36 hours (A), 10 (B) and 20 (C) postpartum days. All animals are positioned such that the skull is at the top of each image. White areas indicate the less dense, air-filled lung and trachea. Thirty-six hours after birth, the lungs of the tammar wallaby are simple and saccular in structure (A). The lung increases in complexity by P10 (B) and further again by P20 (C) with an increase in the number of air sacs and a decrease in the size of these air sacs. Scale bar represents 1 mm.

Thirty-six hours after birth, the lung of the tammar wallaby is a simple saccular structure (Figure 1), which increases markedly in complexity by P10 and further again by P20. This increase in complexity is concurrent with a decrease in the size of air sacs, and at these ages the inflated lung tissue was seen as a speckled intensity pattern, similar to that seen in more developed eutherian species [23], [27].

**Figure 2:**
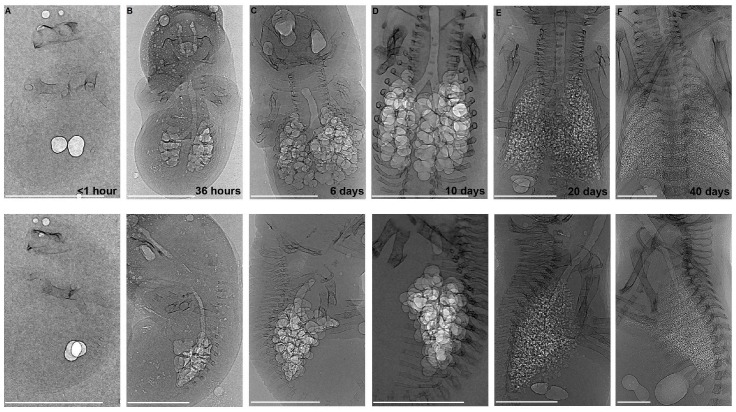
Phase contrast X-ray imaging of the developing fat-tailed dunnart using a synchrotron radiation source. Anterior-posterior (top) and lateral (bottom) views of the developing fat-tailed dunnart from birth (A) to 40 postpartum days (F). White areas indicate the less dense air-filled lung and trachea. Some air may also be noted in the nares (located at the top of the images) or on the body surface. The fat-tailed dunnart undergoes a substantial degree of extra-uterine lung development from just 2 ‘air bubbles’ visible after parturition (A). At P10 (D), the lungs are still characterised by large open circular sacs. By P20 (E) the size, shape and number of air sacs have substantially altered. Scale bar represents 1 mm.

**Figure 3:**
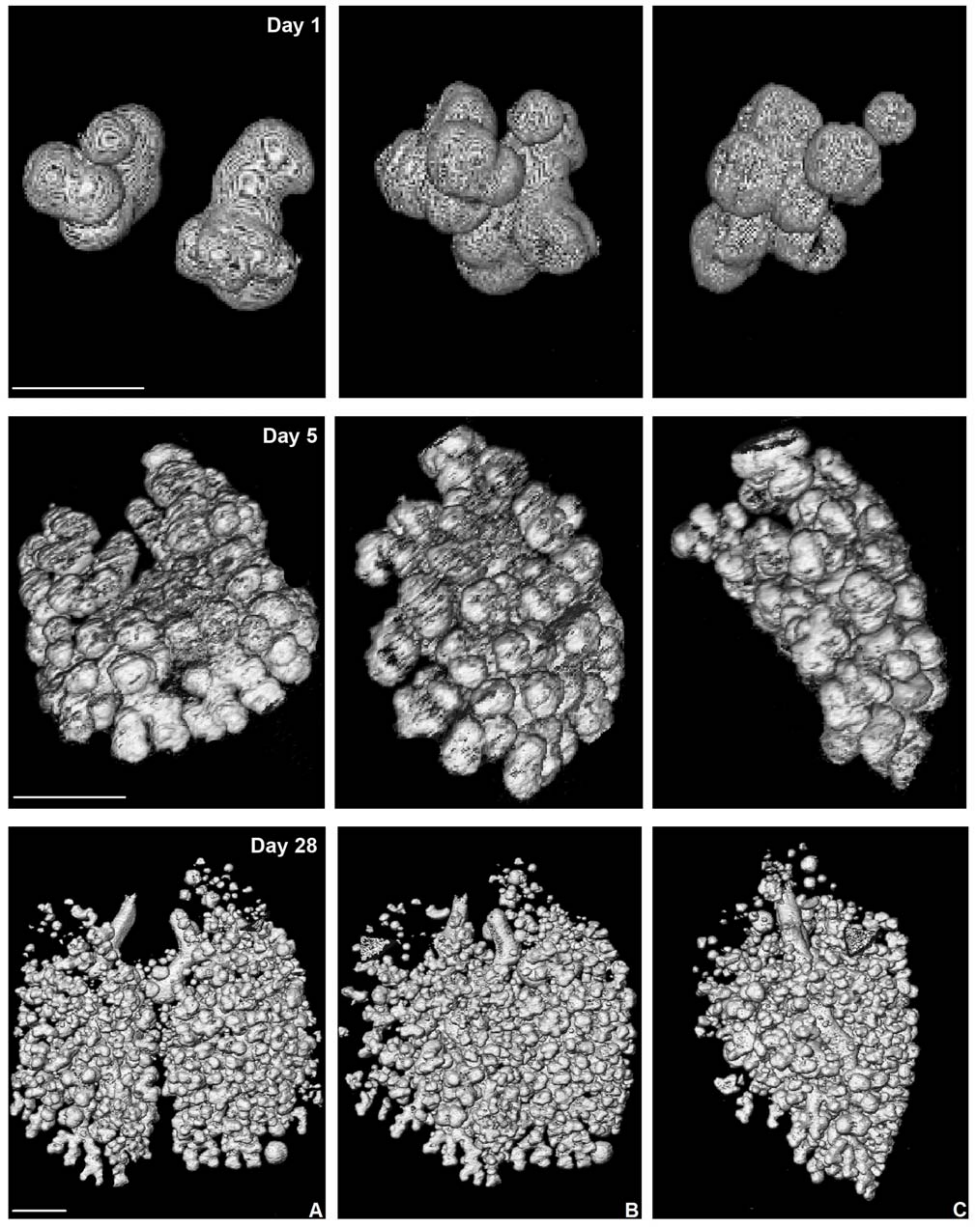
3-Dimensional volume rendering from fat-tailed dunnart computed tomography data sets. Shown are three frames captured over 90 degrees of lung rotation. The series consists of an anterior view (A), a lateral view (C) and the mid-point between them (B), further demonstrating the increase in air sac number, and changes to air sac shape throughout the first weeks of lung development in the fat-tailed dunnart. Scale bar represents 200 µm.

The lungs of the newborn fat-tailed dunnart are more immature than those of the tammar wallaby. The plane radiographs of the developing fat-tailed dunnart (Figure 2) indicate that within the first hour of life, just two lung sacs contain air. Within 36 hours, the dunnart has doubled in body mass, and undergone an increase in the complexity of the lung. It is at this age that air is first observed in the trachea (the trachea otherwise is filled with liquid, pers. obs. Simpson and Frappell), though not in all animals imaged. Further compartmentalisation occurred in the lung between 6 and 10 postnatal days, however, the lung was still observed as large circular air sacs at the end of this period, the shape of which can be better observed in the 3-D rendering (Figure 3). Between 10 and 40 postnatal days, substantial changes were seen in the architecture of the lung (Figures 2 and 3). The air sacs were no longer large circular sacs, and lung volume and surface area were improved through extensive increases in air sac number, as well as architectural complexity.

**Figure 4:**
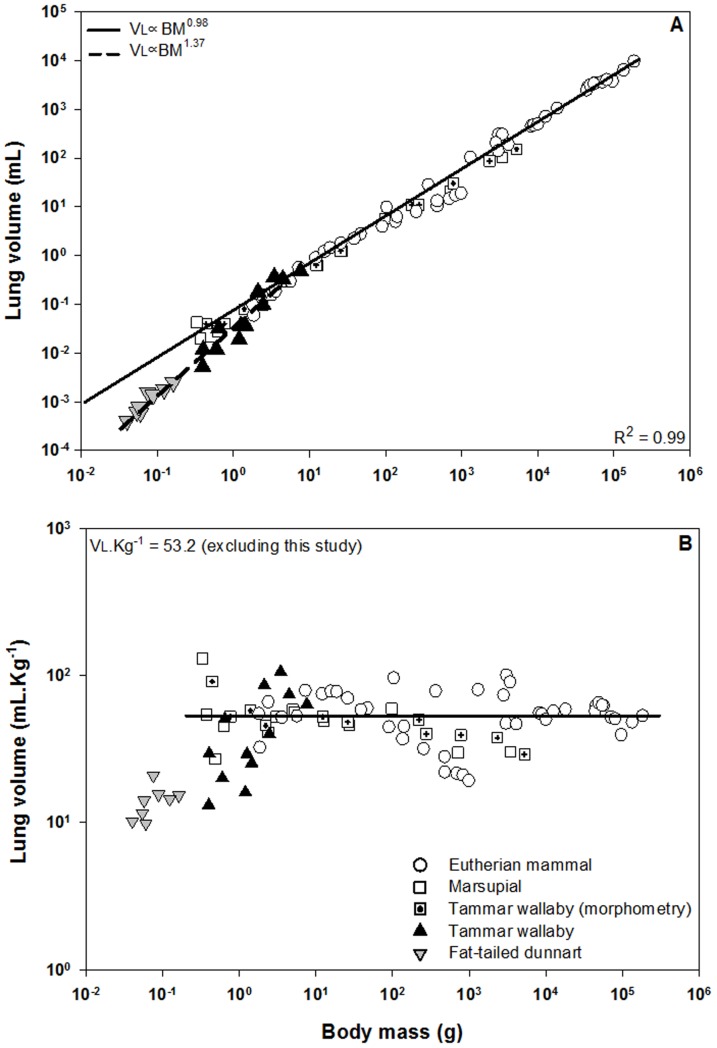
Lung volume (A) and mass specific lung volume (B) as a function of body mass during postnatal development. Open circles represent both adult and newborn eutherian mammals. Open squares represent newborn and adult marsupials, including the tammar wallaby (square containing cross). Black triangles represent tammar wallaby joeys measured in this study aged from birth to P30. Grey inverted triangles represent fat-tailed dunnart pouch young measured in this study from P1–P20. Each triangle represents a single marsupial. The dotted regression line relates to the marsupials in this study while the solid regression line is derived from all other data points. The newborn marsupials measured in this study (with synchrotron imaging) show a lower than expected lung volume, until a body mass greater than 1 g, when the lungs become the main (only) source of gas exchange. Data taken from [2], [10], [31], [38], [42], [43], [45], [46], [47], [48], [49].

**Figure 5:**
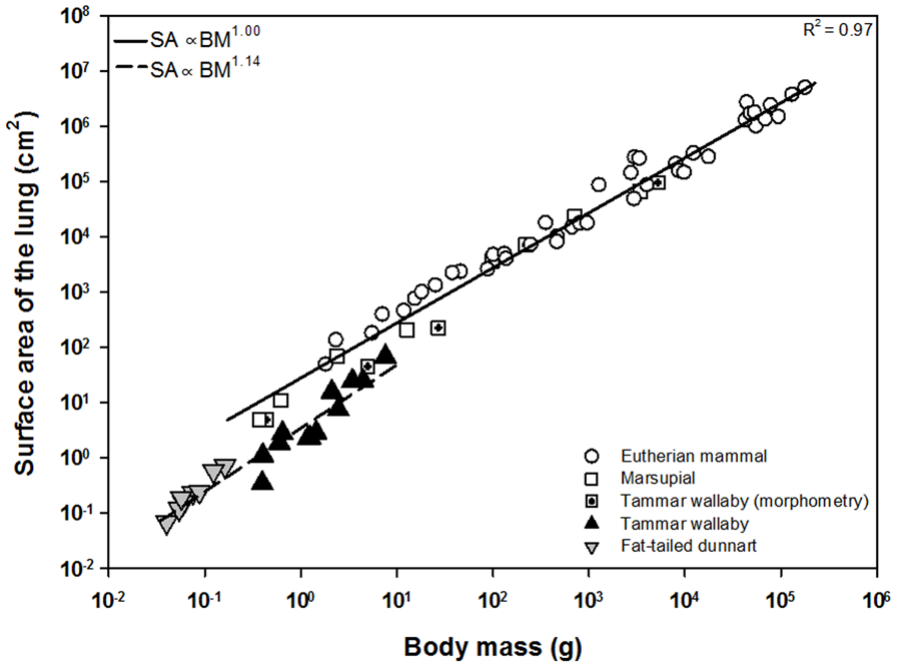
Surface area of the lung as a function of body mass during postnatal development. The air-tissue interface during postnatal lung development of the fat-tailed dunnart and tammar wallaby is less than that predicted from the allometric equation. This is in line with other marsupials, and most likely due to the fact that alveolarisation and the subsequent increase in surface area by septation does not commence until after P70 in the tammar wallaby and P45 in the fat-tailed dunnart. Symbols, lines and references as in Figure 4.

On the first day of postnatal life, the neonatal tammar wallaby and fat-tailed dunnart displayed lower lung volumes and parenchymal surface areas (See Table 1) than were expected from morphometrically determined allometric equations relating these variables to body mass (Figures 4 & 5). The slopes for both lung volume (p<0.001) and surface area (p<0.001) in the newborn marsupials using synchrotron imaging were significantly different from that of the eutherians determined by traditional morphometric techniques. However, lung volume is predicted to scale with mass as expected after the neonatal marsupial reaches a body mass of ∼1 g and no longer relies on the skin for gas exchange (Figures 4 & 5).

**Table 1:** Lung volume and surface area on the first day of postnatal life in the tammar wallaby and fat-tailed dunnart, calculated from computed tomography datasets obtained using a synchrotron radiation source.

	*N*	*Age (hours)*	*Mass (mg)*	*Lung volume (µL)*	*Surface area (cm^2^)*
Tammar wallaby	3	8.3 (13.6)	465 (114)	9.672 (3.861)	1.115 (0.765)
Fat-tailed dunnart	1	6	16	0.41	0.069

Data for the tammar wallaby is expressed as mean (standard deviation).

## Discussion

It is generally accepted that viability of the newborn mammal depends on an adequately developed respiratory system. Indeed, the neonate must rapidly adapt to air breathing through clearance of fetal lung liquid, the onset of a rhythmic breathing pattern and establishment of a functional residual capacity [30]. However, the increasing evidence that newborn marsupials rely to varying degrees on their skin for gas exchange [1], [6], [9] queries the need for a viable respiratory system at birth and questions whether the short gestation common in marsupials is sufficient to allow adequate development of the respiratory system prior to birth.

In the 1 hour old dunnart, the two ‘air bubbles’ observed indicate that either the lung is grossly under-developed and is comprised of only two simple sacs or, alternatively, the architecture of the lung is more developed, but the airspace not completely cleared of fetal lung liquid, rendering the phase contrast non-existent in these fluid filled areas of the lung. In fact, the latter explanation is plausible given that light and electron microscopy demonstrated a thick septal wall within the primitive tubular structures and an over-abundance of surfactant in the airspaces [1]. The predominant driver for airway liquid clearance is trans-pulmonary hydrostatic pressure, generated by inspiratory activity [21], and since the newborn fat-tailed dunnart rarely demonstrates inspiratory activity [1] and both species rely on their skin to supplement gas exchange until a body mass of 1 gram, it could be argued that clearance takes place over an extended period of time as the respiratory rhythm is established. The impact of a delay in lung liquid clearance, combined with the immature architecture of the airways, will be low lung volumes and decreased surface area leading to inefficient gas exchange.

The lung volumes determined in the present study represent the air-filled regions of the lungs (exclusive of the upper airways), not the morphological volumes determined in previous studies with traditional microscopic techniques. The calculation of air-filled volumes may over-estimate the functional or physiological lung volumes as they will undoubtedly include regions where gas exchange is limited; for example, larger airways and regions of the lung where thick interstitium and/or poor capillarisation prevent efficient gas exchange; see [1]. Regardless, the air-filled lung volume determined in the newborn marsupial, whether equal to or greater than the physiological lung volume, is less than that predicted from allometric equations for eutherian newborns that rely entirely on the lungs for gas exchange (Figure 4). Given a reduced lung volume, it is perhaps not surprising that marsupial neonates are reliant on their skin to varying extents for gas exchange. The lung volume of the 1 g tammar wallaby neonate reaches values expected for a eutherian of its size, which coincidently is the time when the lung is responsible for 100% of gas exchange [6]. We currently have no data for dunnarts above 20 days of age (0.18 grams).

The low lung volume at birth in marsupials may relate to the stage of lung development; the earlier the stage the lower the volume and the greater the extent of cutaneous gas exchange. Microscopic morphology has determined that lung volume increases ∼23 fold through development in humans and rats, [11], [31], with marsupials demonstrating a much greater increase in lung volume; a 3,800 fold increase in the case of the tammar wallaby [32] and an 8,000 fold increase in the quokka [10]. In the quokka, dramatic increases in lung volume were observed during the first 5 postnatal days as the lung changed from the canalicular to the saccular stage of lung development. This increase was predominantly due to airspace expansion rather than septal development [10] as septal development occurs later. The presence of a well organised capillary bi-layer is indicative of transition to the saccular stage of lung development [33]; a feature which is not observed in the fat-tailed dunnart until between P27 and P45, around the same time that cutaneous exchange ceases to be important [1].

There are numerous consequences to low lung volumes in newborns, including the greater tendency for alveoli (or air sacs) to collapse. Newborns often minimise this potential problem by maintaining a dynamic elevation of FRC above resting lung volume. In many newborns, this is achieved by a slowing of expiratory emptying (by braking action of the inspiratory muscles), coupled with narrowing of the glottis during expiration, to prolong the expiratory time constant [34], [35]. This strategy is pronounced in the newborn marsupial, where a characteristic post-inspiratory pause has been reported in the breathing trace of all marsupial neonates studied to date [6], [7], [36], [37]; the breath-hold achieved by a glottal closure at the end of inspiration. Dynamic elevation of FRC not only serves to overcome lung collapse it also increases the opportunity for gas exchange and minimises the energetic cost of breathing when the mechanical properties of the respiratory system do not favour efficient ventilation [9].

It is possible that the lung volumes reported in this study are lower than the functional lung volumes found in living neonates due to the lungs deflating below FRC at death. In addition, the possibility of diffusion of air from the lung into the vascular compartment and neighbouring tissues, and consequent underestimation of lung volume, during the preservation of animals must be considered as a potential limitation in this study. In contrast, morphometric estimates of lung volumes and surface areas are sometimes performed after fixation of the lungs to a given tracheal pressure; previous studies in the neonatal tammar wallaby have used 20 cm H_2_O [32], [38]. Filling the lung with fixative to a tracheal pressure of 20 cm may lead to an overestimation of lung volume and surface area due to the highly compliant lung and chest wall of these newborns [39], particularly since lungs inflated with liquid have a much larger compliance and are easier to distend than air-filled lungs [40]. Alternatively, other studies with marsupial neonates have totally immersed the neonate after severing the head to allow fixative inflow [12], hence avoiding the issue of high pressure over-inflation of the lung. Regardless of the preservation method, we have confidence in the robustness of the calculations of lung volumes, with comparable lung volumes reported for the tammar wallaby from a body mass of 1 gram when there is no longer a reliance on the skin to support respiration (figure 4). In addition, others have demonstrated the comparability of lung volumes and surface areas derived from more conventional computed tomography data sets with histological estimations [41].

The surface area of the lung available for gas exchange in the wallaby and dunnart is also below values predicted from allometry (Figure 5), though is in line with previous studies of surface areas using traditional morphometric techniques in marsupials [12], [32]. Lung development involves periods of tissue proliferation and periods of expansion [42]. In contrast to eutherian neonates, marsupial pouch young do not have the high energetic cost associated with thermoregulation and therefore have lower oxygen requirements until the attainment of endothermy. Previous ultrastructural studies have shown that the development of the airways and blood vessels occurs at the expense of parenchymal development during the ectothermic period of the tammar wallaby [38]. Indeed, such studies have shown that changes in the lung up to 20 days are due largely to expansion of the air spaces while tissue proliferation and air sac subdivision is most pronounced during the transitional period from ectothermy to endothermy (after 70 postnatal days, ∼100 g) [38], with true enothermy being attained around 200 days, ∼300 g [7]. These observations support the lower surface areas observed in this study during the early neonatal period and the slow shift towards the allometric line predicted for endothermic eutherians (Figure 5). In addition, marsupials are born with lungs pre-alveolarisation, and it is not until this phase of lung development that we would anticipate significant increases in alveolar surface area as a result of secondary septation. From birth to P70, the lungs of the tammar wallaby are in the saccular stage of lung development [43], while the fat-tailed dunnart does not commence alveolarisation until at least P45 [1]. The mere presence of septation is not indicative of an increase in functional surface area for gas exchange. In fact, it is estimated that only about 50% of the surface area of the terminal air sac in the newborn tammar wallaby, which is well developed when compared to the quokka and fat-tailed dunnart, is actually available to exchange gas due to placement of the blood vessels [43].

The extensive lung development observed in the weeks after birth is not surprising given an increasing reliance on the lung for gas exchange. At the same time, there is a decrease in cutaneous surface area with respect to body size and an increase in the demand for oxygen, particularly as the capability for thermogenesis develops [44]. Low lung volumes during early postnatal life, delayed fluid clearance from the airways and mechanical distortion, together with the absence of a continuous breathing pattern at birth in the smallest of the mammalian newborns all point towards constraints on the respiratory system. It remains to be elucidated whether the neuromuscular machinery required to drive ventilation is present in the smallest marsupial newborns. While quantification of surface area, lung volume, capillary density and thickness of the air-blood barrier using traditional morphometric techniques would have been desirable, pressure fixation was not possible in these minute newborns.

In summary, phase contrast imaging using a synchrotron radiation source confirmed that marsupials such as the fat-tailed dunnart and the tammar wallaby are born with structurally immature lungs when compared to eutherian mammals and underwent marked increases in architectural complexity during the postnatal period. In addition, these marsupials had lower lung volumes and parenchymal surface areas than were expected from morphometrically determined allometric equations relating these variables to body mass during the early neonatal period. These findings further support the maxim that cutaneous gas exchange occurs in the marsupial neonate because the respiratory apparatus is not yet capable of meeting the gas exchange requirements of the newborn.
